# Mathematical analysis to prioritise strategies for malaria elimination

**DOI:** 10.1016/j.jtbi.2018.07.007

**Published:** 2018-10-14

**Authors:** Nakul Chitnis, Allan Schapira, Christian Schindler, Melissa A. Penny, Thomas A. Smith

**Affiliations:** a Swiss Tropical and Public Health Institute, Socinstrasse 57, Basel 4002, Switzerland; b University of Basel, Basel 4003, Switzerland

**Keywords:** Eradication, Mathematical model, Economic model, Health burden

## Abstract

Malaria and some other tropical diseases are currently targeted for elimination and eventually eradication. Since resources are limited, prioritisation of countries or areas for elimination is often necessary. However, this prioritisation is frequently conducted in an *ad hoc* manner. Lower transmission areas are usually targeted for elimination first, but for some areas this necessitates long and potentially expensive surveillance programs while transmission is eliminated from neighbouring higher transmission areas. We use a mathematical model to compare the implications of prioritisation choices in reducing overall burden and costs. We show that when the duration of the elimination program is independent of the transmission potential, burden is always reduced most by targeting high transmission areas first, but to reduce costs the optimal ordering depends on the actual transmission levels. In general, when overall transmission potential is low and the surveillance cost per secondary case compared to the cost per imported case is low, targeting the higher transmission area for elimination first is favoured.

## Highlights

• Malaria and many other diseases are currently targeted for elimination.
• Prioritisation of areas for elimination often occurs in an *ad hoc* manner.
• In low transmission areas, prioritising higher transmission site reduces total burden.
• In low transmission areas, prioritising higher transmission site reduces total costs.
• In high transmission areas, prioritisation requires more detailed analysis.

## 1 Introduction

The *Global Technical Strategy for Malaria 2016–2030*, released by the World Health Organization (WHO) (World Health Organization, 2015), sets the current global goals for malaria control and elimination by 2030 as: (i) reducing the number of malaria cases and deaths globally by 90% as compared to 2015; and (ii) eliminating and preventing re-establishment of transmission in at least 35 countries where malaria transmission was ongoing in 2015. The main strategies towards achieving these goals are (i) “control through universal access to malaria prevention, diagnosis and treatment”; (ii) intensifying efforts towards elimination and prevention of re-introduction; and (iii) “transforming malaria surveillance into a core intervention” of both control and elimination strategies (World Health Organization, World Health Organization).

Malaria control activities are recommended in all locations where transmission persists (although it is sometimes not deployed in locations where financial and/or operational resources are insufficient). However, efforts to eliminate malaria are mainly focused on the fringes of its geographical range, for example in the Asia-Pacific region (Gosling et al., 2012) and in Southern Africa (Southern African Development Community, 2013).

There is a global health priority in eliminating foci of drug resistance in the Greater Mekong subregion (Gueye et al., 2014), and spatially progressive elimination may be rational where the risk of re-establishment of transmission is low (Lines, Schapira, Smith, 2008, Smith, Cohen, Chiyaka, Johnston, Gething, Gosling, Buckee, Laxminarayan, Hay, Tatem, 2013), or where a small focus of transmission has a disproportionate economic importance. Targeting isolated islands and other areas with low transmission potential for malaria elimination may also have value as tests of new technologies or systems; but in general it is unclear whether targeting low transmission areas is a better strategy than focusing those resources on eliminating malaria from higher transmission areas, especially when these lower transmission areas face risks of malaria importation from neighbouring higher transmission areas.

The strategy of progressive elimination from the fringes has been criticised because of the implicit inequity of prioritising low burden areas (Shah, 2010). This strategy also ignores the important lesson from those programs that have been successful in eradicating a disease (smallpox (Henderson, 2009), or that have approached eradication (polio (Aylward et al., 2003) and dracunculiasis (Ruiz-Tiben and Hopkins, 2006), that eradication programs need to focus early in challenging areas which are likely to remain a threat after the disease is gone elsewhere. More generally, it seems likely that any eradication or elimination program will be more efficient if core areas that export the infection are targeted at the start. This concurs with the experience of countries that have eliminated or approached elimination. Iran is now on its second attempt at national malaria elimination. Each time, interventions were rolled out nationally, burden reduction in the high transmission south east has been key to near-elimination in the north and west (Hemami et al., 2013).

An effective surveillance system is a key constituent intervention of intensified control efforts to eliminate malaria; and remains essential after malaria has been eliminated to prevent re-establishment of transmission (Kelly, Tanner, Vallely, Clements, 2012, World Health Organization). It must include a reactive component that is effective in detecting imported cases and preventing onward transmission from them. The surveillance system will need to be maintained as long as there is a risk of reintroduction, that is, until malaria has been eradicated. This can be operationally and financially challenging in tropical areas with high vectorial capacity (malaria transmission potential) (Roberts, 2010, Schapira, Zamani, 2012, Smith, Cohen, Moonen, Tatem, Sabot, Ali, Mugheiry, 2011, World Health Organization, Global Malaria Programme, University of California, San Francisco, 2012).

There is therefore a need for decisions concerning elimination to be based on criteria that consider costs, overall disease burden and the risks associated with different options. This paper aims to provide a mathematical formulation to guide strategic thinking about how zones with different levels of disease burden should be targeted for elimination, on the assumptions that (i) malaria control is maintained in all areas; (ii) elimination is technically possible in all the areas being considered; (iii) but resources to intensify control programs to achieve elimination are limited to targeting one area at a time. The results are expressed in terms of general principles that may be applicable at different spatial scales across the whole range of malaria transmission intensities. To derive these principles, we only consider a simple economic model of two areas of equal population here, but some of this analysis may be extended to multiple areas. Although many studies, modelling and otherwise, have investigated the feasibility of malaria elimination (Moonen et al., 2010), and the persistence of elimination (Chiyaka, Tatem, Cohen, Gething, Johnston, Gosling, Laxminarayan, Hay, Smith, 2013, Smith, Cohen, Chiyaka, Johnston, Gething, Gosling, Buckee, Laxminarayan, Hay, Tatem, 2013), none have considered such mathematical economic models for ordering areas for intensified control efforts to achieve elimination.

We consider a simple system consisting of two connected geographical areas with similar populations but different initial levels of transmission, and equal (symmetric) movement of in both directions through the short term movement of people and possibly mosquito vectors. Symmetrical movement is a reasonable assumption here because imported malaria cases are usually not due to immigration but due to the short term movement of visitors from areas with higher transmission or returning residents. Therefore we assume that the importation of infection in either direction depends only on the prevalence in the source population.

Both areas are initially under control, with current tools, such as long lasting insecticidal nets and indoor residual spraying, maintaining the annual disease burden in each area at a relatively constant level. Although there are likely to be seasonal variations within each area, we do not explicitly consider them here because we are more interested in the general principles of the relationship between importation and elimination (which do not depend on seasonality), and not in the details of planning such elimination strategies (which depend on seasonality).

We assume that the technologies for time-limited elimination throughout the system are available (technical feasibility) with additional tools such as reactive case detection and reactive vector control (Moonen, Cohen, Snow, Slutsker, Drakeley, Smith, Abeyasinghe, Rodriguez, Maharaj, Tanner, Targett, 2010, World Health Organization). However, the human and/or financial resources to apply these additional intervention measures in both areas simultaneously are not available, so that overall elimination can only be achieved by intensified control measures in one area at a time, in each case until local transmission is interrupted.

We label the higher transmission site as i=1, with transmission potential (measured by the reproduction number) *R*_1_ (see Table 1). We label the lower transmission site as i=2 with transmission potential *R*_2_ where *R*_2_ < *R*_1_. Both sites are additionally characterised by malaria prevalence at equilibrium, *p_i_*, annual disease burden, *B_i_*, vulnerability, *V_i_*, and required duration of intensified control to eliminate transmission, *T_i_*. More detailed descriptions of these and all other parameters are provided in Table 1. We define elimination here as the lack of sustained local transmission (that is, imported cases may lead to a few secondary cases but each chain of infection dies out and malaria cannot reestablish itself in the population). We use the WHO definition of elimination as the “interruption of local transmission (reduction to zero incidence of indigenous cases)” (World Health Organization, 2016). WHO malaria terminology distinguishes between introduced cases (“first generation local transmission” from an imported case) and indigenous cases (“contracted locally with no evidence of importation and no direct link to transmission from an imported case”) (World Health Organization, 2016). Re-establishment of transmission is defined as the “renewed presence of a measurable incidence of locally acquired malaria infection due to repeated cycles of mosquito-borne infections in an area in which transmission had been interrupted” (World Health Organization, 2016). Therefore in a state of elimination, local transmission is possible so secondary (introduced) cases may arise from an imported case, but these should not lead to sustained (re-established) transmission.

**Fig. 1:**
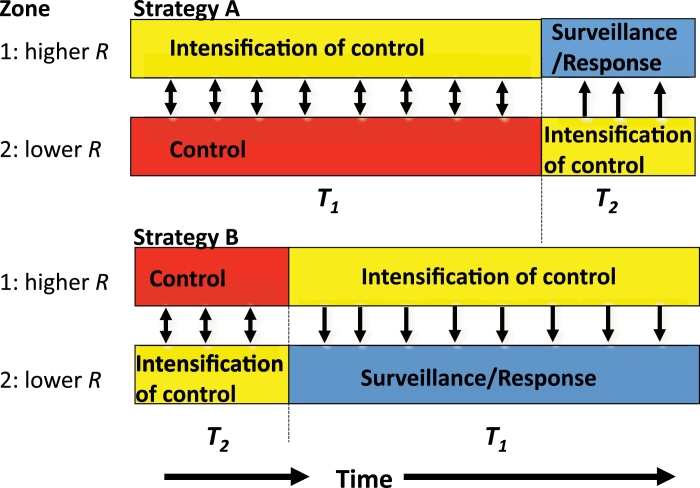
Strategies being compared. The black arrows indicate the direction of the export/import of infected cases. Here, *R* denotes the transmission potential and *T_i_* denotes the duration of intensified measures needed to achieve elimination (Table 1).

There are two possible strategies for elimination across both sites: J=A, intensification of control in the higher transmission site (i=1) first, with continued control in lower burden area; and J=B, intensification in the lower transmission site (i=2) first, with continued routine control in the higher burden area. These two strategies are illustrated in Fig. 1.

**Table 1:** Notation and definitions. Here *i* denotes the site (where *i* ∈ {1, 2} and i=1 represents the higher transmission site), and *J* denotes the strategy (where *J* ∈ {A, B} and J=A represents the strategy of targeting the higher transmission site first).

	Description	Dimensions
*R_i_*	Average transmission potential in area *i* before intensified control measures. Transmission potential is the control reproduction number, sometimes denoted as *R_c_*, (Smith et al., 2009).	Dimensionless
*p_i_*	Equilibrium prevalence in source area *i* (before intensified elimination measures).	Proportion
*B_i_*	Pre-elimination burden of disease per unit of time in area *i*.	Disease events / (person time)
*b_i_*	Average burden of disease per unit time during intensified elimination program in area *i*.	Disease events / (person time)
*B*_max _	Maximum possible burden.	Disease events / (person time)
*ψ_B_*	Saturation factor for burden.	Dimensionless
*V_i_*	Vulnerability of area *i* following elimination from area *i* only. Vulnerability is defined as either proximity to a malarious area or frequent influx of infected humans and/or infective mosquitoes (World Health Organization, 2012). Here we assume vulnerability is equivalent to the rate of imported infections.	Infections / (person time)
*η*	Extent of migration of humans and/or mosquitoes between the populations.	1 / (person time)
*V*_max _	Maximum possible vulnerability.	Infections / (person time)
*ψ_V_*	Saturation factor for vulnerability.	Dimensionless
*T_i_*	Duration of intensified measures needed to achieve elimination in area *i*.	Time
*T*_max _	Maximum possible duration of intensified measures needed to achieve elimination.	Time
*ψ_T_*	Saturation factor for time to elimination.	Dimensionless
*E_J_*	Total burden of disease over the whole program, conditional on strategy *J*.	Disease events / person
*ΔE*	Net health benefit of strategy A (i.e., the burden averted relative to the alternative strategy B).	Disease events / person
*S_J_*	Total surveillance costs, conditional on strategy *J*.	Currency
*S*_0_	Fixed cost of a surveillance system.	Currency
*β*	Health systems cost per primary imported case.	Currency
*γ*	Health systems cost per secondary case.	Currency
*C_J_*	Total cost of strategy *J*.	Currency
*ΔC*	Cost saving with strategy A compared with strategy B.	Currency
*λ*	Ceiling ratio: the amount of money that the program is willing to pay to avert disease.	Currency / (disease event)
*M*	Net monetary benefit of strategy A.	Currency

We note that the intensification program must include an effective surveillance response system. After the intensification program has eliminated malaria in the first area and has moved to the second area, the surveillance response system must continue to operate in the first area to prevent reintroduction (re-establishment of transmission), but other control interventions may be withdrawn. We therefore assume that after malaria elimination in the first transmission zone, the transmission potential for secondary cases remains at pre-intervention levels, but there is an effective surveillance system, that prevents re-establishment of transmission from these secondary cases.

## 2 Assumptions of transmission potential

Determining the preferred strategy to minimise overall disease burden or costs requires additional assumptions about the relationships of the transmission potential, *R*, with the annual disease burden, *B*, the vulnerability, *V*, and the required time for intensified control measures to interrupt transmission, *T*. These three relationships in turn depend on the relationship between the transmission potential, *R*, and the equilibrium prevalence, *p*, during the control phase.

### 2.1 Relationship between prevalence and transmission potential

The general form of the relationship of the equilibrium prevalence to the reproduction number is different in different models of malaria transmission. When transmission potential is low, infections are sporadic; therefore immunity is negligible, neither sequential nor concurrent co-infections are likely, and simple susceptible-infected-susceptible models (corresponding to the original model of Ross, 1916) represent a reasonable approximation to malaria dynamics. In this case, *p_i_* in the stable state relates to reproduction number via

(1) $$p_{i}=1-\frac{1}{R_{i}},$$

with derivative

(2) $$\frac{dp_{i}}{dR_{i}}=\frac{1}{R_{i}^{2}}.$$

**Fig. 2:**
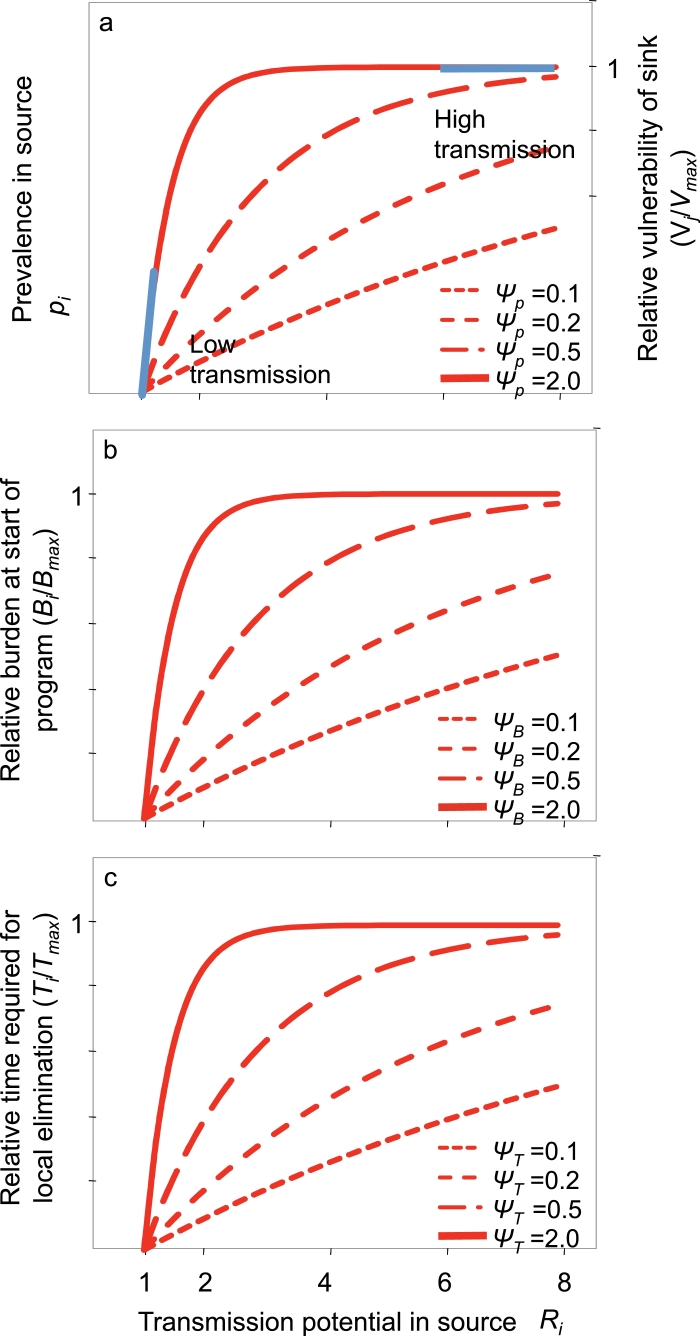
Proposed relationships of equilibrium prevalence, vulnerability, disease burden and duration of intensified measures needed to achieve elimination with transmission potential. a) Prevalence and vulnerability: vulnerability is proportional to prevalence in the complementary population so they are represented with the same curves. The blue lines correspond to the extreme cases analysed in detail. b) Disease burden. c) Duration of elimination program. (For interpretation of the references to color in this figure legend, the reader is referred to the web version of this article.)

It follows that, in the limiting case of very low transmission, $R_{i}=1,$

(3) $$\frac{dp_{i}}{dR_{i}}\approx 1,$$

implying that *p_i_* is approximately directly proportional to *R_i_* with an offset of 1 (Fig. 2(a)) and gradient 1, i.e. $p_{i}\approx R_{i}-1$. In high transmission settings where immunity and superinfection play important roles, prevalence saturates at some level, *p*_max _, that depends on the extent of transmission heterogeneity but is independent of *R_i_*. Although the value of *R_i_* at which *p_i_* saturates depends on transmission heterogeneity and the dynamics of acquired immunity, the general shape of the relationship between *p_i_* and *R_i_* can be approximated with curves of the form,

(4) $$p_{i}=p_{\max}\left(1-\exp\left(-\psi_{P}\left(R_{i}-1\right)\right)\right),$$

where the saturation factor, *ψ_P_*, is a positive constant (Fig. 2(a)). This assumption fits well with analysis of data relating malaria prevalence to the entomological inoculation rate across different sites in Africa, which considered such an exponential curve amongst others (Smith et al., 2005).

### 2.2 Relationship between annual disease burden and transmission potential

On the assumption that the populations are initially close to a steady state, pre-elimination annual disease burden, *B_i_*, is related to prevalence, *p_i_*, and hence to *R_i_* by some monotonic function, $B_{i}=f_{B}\left(R_{i}\right)$. Since *R*_1_ > *R*_2_, it follows that *B*_1_ > *B*_2_ and one set of analyses considers the inferences that can be drawn without making any stronger assumptions about transmission-burden relationships (Table 2). In addition, we consider the inferences that follow from assuming that pre-elimination disease burden saturates as transmission potential increases, following the relationship,

(5) $$B_{i}=B_{\max}\left(1-\exp\left(-\psi_{B}\left(R_{i}-1\right)\right)\right),$$

where *B*_max _ and *ψ_B_* are two positive constants defining the relationship between burden and transmission potential, analogous to the relationship between prevalence and transmission potential (Fig. 2(b)).

**Table 2:** Scenarios modelled and results for analysis of burden.

	$T_{1}=T_{2}=T$: *Fixed duration of intensified measures to reduce incidence to negligible levels in area i*	*T*_1_ > *T*_2_: *Duration increases with transmission*
*B*_1_ > *B*_2_: *Burden increases with transmission*	Strategy A minimises overall burden.	Strategy A minimises overall burden if and only if *B*_1_/*B*_2_ > *T*_1_/*T*_2_.

### 2.3 Relationship between vulnerability and transmission potential

*V_i_*, the vulnerability of sink population, *i*, to source population *j* (for *j* ∈ {1, 2}, *j* ≠ *i*), depends on the rate at which infections are exported from the source, which is proportional to the prevalence in the source population, *p_j_. p_j_* is a function of *R_j_*, implying that *V_i_* can also be expressed as a similar function of *R_j_*: $V_{i}=\eta p_{j}=f_{V}\left(R_{j}\right),$ where the constant of proportionality *η* captures the extent of migration of humans and/or mosquitoes between the populations.

In low transmission settings, vulnerability, like prevalence, is proportional to the transmission potential of the source patch,

(6a) $$\begin{array}{cc} V_{1} & \approx \eta \left(R_{2}-1\right), \end{array}$$

(6b) $$\begin{array}{cc} V_{2} & \approx \eta \left(R_{1}-1\right). \end{array}$$

At high transmission, vulnerability, like prevalence, saturates so that if both areas are at high transmission, then $V_{1}=V_{2}=V$ (Table 3).

**Table 3:** Scenarios modelled and results for analysis of costs.

	$T_{1}=T_{2}=T$: *Fixed duration of intensified measures to reduce incidence to negligible levels in area i*	*T*_1_ > *T*_2_: *Duration increases with transmission*
$V_{i}=\eta \left(R_{j}-1\right)$ for *i, j* ∈ {1, 2} and *i* ≠ *j: Low transmission*	Strategy A has lower cost	Strategy A has lower cost
$V_{1}=V_{2}$: *High transmission*	Strategy B has lower cost	As *R*_2_ increases, strategy A is more likely to have lower cost; as *R*_1_ increases, strategy B is more likely to have lower cost

Similar to the relationship between *R* and *p*, the general shape of the relationship between *V* and *R* can be approximated with curves of the form,

(7a) $$\begin{array}{cc} V_{1} & =V_{\max}\left(1-\exp\left(-\psi_{V}\left(R_{2}-1\right)\right)\right), \end{array}$$

(7b) $$\begin{array}{cc} V_{2} & =V_{\max}\left(1-\exp\left(-\psi_{V}\left(R_{1}-1\right)\right)\right), \end{array}$$

where *V*_max _ and *ψ_V_* are two positive constants defining the relationship between vulnerability and transmission potential, and where

$${\frac{dV_{1}}{dR_{2}}|}_{V_{1}=0}={\frac{dV_{2}}{dR_{1}}|}_{V_{2}=0}=\eta =V_{\max}\psi_{V},$$

with the low and high transmission examples described above represent limiting cases (Fig. 2(a)).

### 2.4 Relationship between duration of intensified efforts to interrupt transmission and transmission potential

The duration over which intensified measures must be sustained to interrupt transmission depends on multiple factors affecting intervention effectiveness. The present analyses consider only the effect of transmission potential, *R*, so that $T_{i}=f_{T}\left(R_{i}\right),$ and the areas are treated as equivalent in other respects.

During the Global Malaria Eradication Programme (GMEP) era from 1955 to 1969, it was assumed that an initial attack phase would comprise of maximal scale up of vector control interventions to interrupt transmission (Pampana, 1963); and that this would only need to be sustained for the natural lifetime of the residual infections, which is approximately independent of the transmission rate, (Macdonald and Göckel, 1964). Therefore, the simplest set of assumptions about the required duration of the elimination measures (Tables 2 and 3) is that they are equal in the two sites,

$$T_{1}=T_{2}=T.$$

However, it is possible that the required duration of the elimination measures is longer in higher transmission settings, so that *T* increases with *R* and *T*_1_ > *T*_2_. This assumption underlies a further set of analyses (Tables 2 and 3). In general, as intervention programs are prolonged and intensified, the environmental drivers of malaria transmission (i.e. the determinants of *R*) may become less important as determinants of program outcomes, relative to operational effectiveness of control programs (see e.g., Mabaso et al., 2007), so that it is to be expected that the function relating *T* to *R* will saturate, like those relating *B* and *V* to *R*, and hence can be approximated with

(8) $$T_{i}=T_{\max}\left(1-\exp\left(-\psi_{T}\left(R_{i}-1\right)\right)\right),$$

where *T*_max _ and *ψ_T_* are two positive constants defining the relationship between the required duration of the elimination program and transmission potential (Fig. 2(c)).

## 3 Analysis

### 3.1 Burden of disease

The total burden of disease, *E_J_*, experienced with strategy *J*, is the sum of the burden during the intensified control phase in the first area to experience intensified control, the pre-elimination burden in the other area, and the burden during intensified control in the second area. For strategy A this is (see Table 1 for notation)

(9) $$E_{A}=\left(b_{1}+B_{2}\right)T_{1}+b_{2}T_{2}.$$

Similarly, the burden experienced with strategy B is

(10) $$E_{B}=\left(b_{2}+B_{1}\right)T_{2}+b_{1}T_{1}.$$

The health benefit of the decision for strategy A is

(11) $$\begin{array}{cc} \Delta E & =E_{B}-E_{A}\\ & =B_{1}T_{2}-B_{2}T_{1}, \end{array}$$

where strategy A minimises burden if *ΔE* > 0. We determine which strategy minimises overall burden, assuming that *B*_1_ is always greater than *B*_2_, for different assumptions about the dependence of *T_i_* on transmission as indicated in Table 2.

In the special case of $T_{1}=T_{2}=T,$ corresponding to a fixed duration of intensified measures as sometimes achieved by the initial GMEP strategy (Pampana, 1963), there is always a health benefit in following strategy A (from Eq. (11) because *B*_1_ > *B*_2_ — details of this analysis are shown in Appendix B). The health effects are maximised if the program starts in the highest burden population and proceeds in order of decreasing burden. This result generalises to multiple populations, and follows from the assumption that transmission potential is an external, unchanging, factor. This contrasts with models for control of epidemics of directly transmitted disease (Rowthorn et al., 2009) where the default assumption of equal transmission potential in different areas leads to an inverse relationship between susceptibility and initial level of transmission, so that prioritisation of the initially lower transmission area minimises overall burden.

If the duration of intensified measures varies with transmission potential, *T*_1_ > *T*_2_, then Eq. (11) implies that the preferred strategy for minimising the overall burden depends on how the ratio of time to elimination relates to the ratio of pre-elimination burden. Strategy B has lowest overall burden if *T*_1_/*T*_2_ > *B*_1_/*B*_2_, while strategy A has lowest overall burden if *B*_1_/*B*_2_ > *T*_1_/*T*_2_. Details of this analysis are shown in Appendix C. In summary, to minimise burden over the total duration of the elimination program, if area 1 has a higher burden than area 2,then it is rational to prioritise elimination efforts in area 1, unless the time it would take to achieve elimination in area 1 is *disproportionately* long compared to the time required in area 2.

While at least the relative values of *B_i_* should be known, this is rarely the case for *T_i_*. We therefore represent the general relationships between burden and duration of intensified control with transmission potential, by the models of Eqs. (5) and (8), respectively. Substitution of these formulae into Eq. (11) indicates that strategy A minimises overall burden if and only if *ψ_T_* > *ψ_B_*, that is, if the time to elimination saturates faster with the reproduction number than does the initial burden. Details of this analysis are shown in Appendix D.

### 3.2 Costs

We assume that the overall costs of intensified measures of either strategy are incurred irrespective of the ordering of areas and duration of each phase (implying that the time scales for elimination occur faster than any secular changes such as socio-economic development that would reduce transmission potential over time). Ignoring discounting, these costs therefore cancel in the formulae for the differences in costs between strategies, and the only differences depend on the surveillance costs (see Table 1 for notation),

(12) $$\begin{array}{cc} \Delta C & =C_{A}-C_{B}\\ & =S_{A}-S_{B}, \end{array}$$

where strategy A is cost-saving if *ΔC* < 0.

The total surveillance costs in each area depend on population size, vulnerability, receptivity, and the duration of the program and can be treated as the sum of three components: the per capita base costs of setting up the system of surveillance *S*_0_ (which we assume to be similar across the two zones), the marginal cost of identifying and managing all primary (imported) cases, (equal to the product of the cost per imported case, *β*, and the importation rate, *V*), and the costs of responding to any secondary cases (introduced or indigenous), equal to the cost per secondary case, *γ*, multiplied by the importation rate, *V*, and the average number of secondary cases, *R*, per imported case, so that the cost of surveillance with strategy A is

(13) $$\begin{array}{cc} S_{A} & =S_{0}+\beta V_{1}T_{2}+\gamma V_{1}T_{2}R_{1}\\ & =S_{0}+V_{1}T_{2}\left(\beta +\gamma R_{1}\right), \end{array}$$

and with strategy B is

(14) $$S_{B}=S_{0}+V_{2}T_{1}\left(\beta +\gamma R_{2}\right).$$

This leads to incremental cost of strategy A from Eq. (12) of

(15) $$\Delta C=V_{1}T_{2}\left(\beta +\gamma R_{1}\right)-V_{2}T_{1}\left(\beta +\gamma R_{2}\right),$$

which can be expressed as a function of the reproductive numbers,

(16) $$\Delta C=f_{V}\left(R_{2}\right)f_{T}\left(R_{2}\right)\left(\beta +\gamma R_{1}\right)-f_{V}\left(R_{1}\right)f_{T}\left(R_{1}\right)\left(\beta +\gamma R_{2}\right).$$

The condition for strategy A being cost saving is then

(17) $$f_{V}\left(R_{2}\right)f_{T}\left(R_{2}\right)\left(\beta +\gamma R_{1}\right)<f_{V}\left(R_{1}\right)f_{T}\left(R_{1}\right)\left(\beta +\gamma R_{2}\right).$$

Using this inequality, we consider which strategy minimises overall costs for different combinations of assumptions about *T*_1_, *T*_2_, *V*_1_, and *V*_2_ as indicated in Table 3.

#### 3.2.1 Low transmission

In low transmission areas, where vulnerability is proportional to transmission potential, $V_{i}=\eta \left(R_{j}-1\right)$ for *i* ≠ *j*, when the required duration of intensified measures is equal, $T_{1}=T_{2}=T,$ the incremental cost of strategy A from Eq. (15) is

(18) $$\begin{array}{cc} \Delta C & =\eta \left(R_{2}-1\right)T\left(\beta +\gamma R_{1}\right)-\eta \left(R_{1}-1\right)T\left(\beta +\gamma R_{2}\right)\\ & =\eta T\left(\beta +\gamma \right)\left(R_{2}-R_{1}\right), \end{array}$$

which is always negative, implying that strategy A has lowest cost (Table (3)). Details of this analysis are shown in Appendix E.

When the required duration of intensified measures increases with transmission, *T*_1_ > *T*_2_, the incremental cost (Eq. (15)) is

(19) $$\Delta C=\eta \left(R_{2}-1\right)T_{2}\left(\beta +\gamma R_{1}\right)-\eta \left(R_{1}-1\right)T_{1}\left(\beta +\gamma R_{2}\right),$$

which is even more strongly negative than for the case where $T_{1}=T_{2}=T,$ implying that strategy A again has lowest cost (Table 3). Details of this analysis are shown in Appendix F.

#### 3.2.2 High transmission

If both areas have sufficiently high transmission, vulnerability saturates, $V_{1}=V_{2}=V,$ because the risk of infection no longer increases with *R*. If the required duration of intensified measures is equal, $T_{1}=T_{2}=T,$ the incremental cost of strategy A from Eq. (15) is

(20) $$\begin{array}{cc} \Delta C & =VT\left(\beta +\gamma R_{1}\right)-VT\left(\beta +\gamma R_{2}\right)\\ & =VT\gamma \left(R_{1}-R_{2}\right), \end{array}$$

which is always positive (in contrast to the analysis of low transmission), implying that strategy B has lowest cost (Table 3). Details of this analysis are shown in Appendix G.

When the required duration increases with transmission, *T*_1_ > *T*_2_, the incremental cost of strategy A is

(21) $$\Delta C=VT_{2}\left(\beta +\gamma R_{1}\right)-VT_{1}\left(\beta +\gamma R_{2}\right),$$

which is negative (favouring strategy A) if and only if

(22) $$R_{2}>\frac{T_{2}}{T_{1}}\left(R_{1}-\frac{\beta }{\gamma }\left(\frac{T_{1}-T_{2}}{T_{2}}\right)\right).$$

**Fig. 3:**
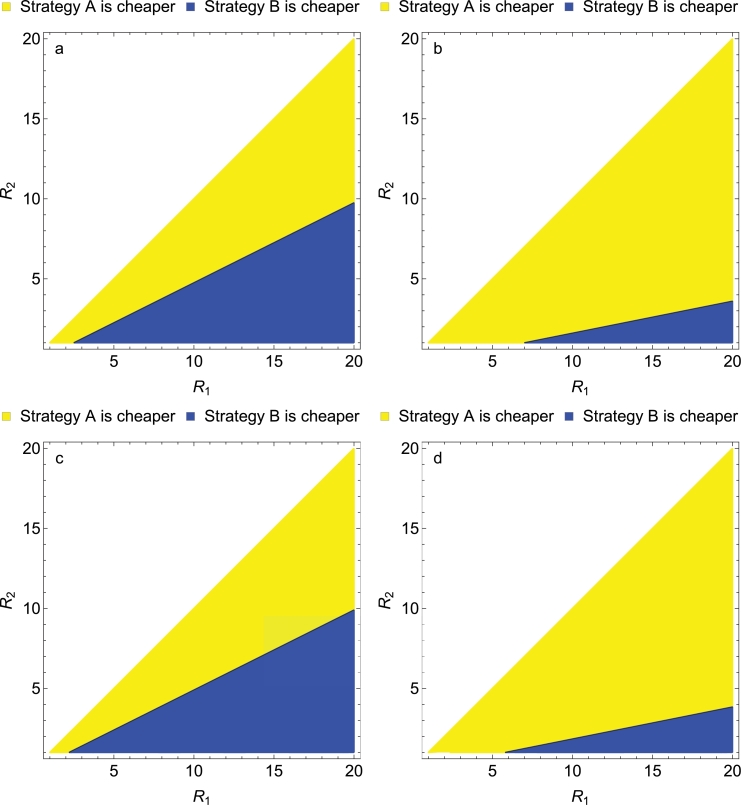
Indifference curves indicating transmission potentials at which the costs are equivalent when time to elimination differs between areas (*T*_1_ > *T*_2_) but vulnerability is saturated ($V_{1}=V_{2}$). The yellow shading indicates the parameter space for which strategy A is cheaper. a) The surveillance costs of a secondary case are twice that of an imported case, γ=2β. The required duration of the elimination program is twice as long in the higher transmission area, $T_{1}=2T_{2}$. b) γ=2β. $T_{1}=5T_{2}$. c) γ=5β. $T_{1}=2T_{2}$. d) γ=5β. $T_{1}=5T_{2}$. (For interpretation of the references to color in this figure legend, the reader is referred to the web version of this article.)

Details of this analysis are shown in Appendix H. Fig. 3 illustrates some of the parts of the parameter space indicating where this condition is satisfied. The parameter values illustrated in Fig. 3(a), in which the surveillance cost per secondary case is twice the cost of a primary case and the time to elimination is twice as high in the higher transmission setting, show a roughly equal division of the reproductive numbers between favouring strategy A and strategy B. Fig. 3(b), in which the time to elimination is five times as high in the higher transmission setting, but with the same surveillance costs as in Fig. 3(a), shows that strategy A is largely preferred to strategy B. Fig. 3(c), in which the surveillance cost per secondary case is five times that of the primary case, but with the same time to elimination as in Fig. 3(a), shows a small increase in situations that favour strategy B when compared to Fig. 3(a) (because strategy A results in a higher ratio of secondary to primary cases than strategy B). Fig. 3(d) shows that when the surveillance cost per secondary case is five times the cost of a primary case and the time to elimination is five times as high in the higher transmission setting, strategy A is largely preferred to strategy B, but there are more settings where strategy B is preferred than in Fig. 3(b). In all four cases, high values of *R*_2_, which are more relevant because vulnerability is more likely to saturate at high transmission potentials, favour strategy A.

#### 3.2.3 Varying transmission with fixed duration of intensified measures

**Fig. 4:**
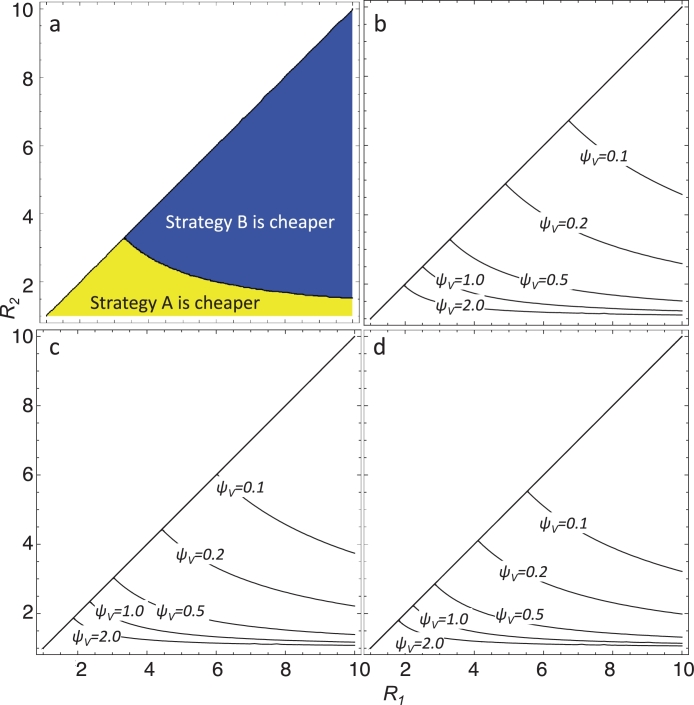
Indifference curves indicating transmission potentials at which the costs are equivalent for the two strategies assuming equal durations of intensified measures in the two sites, $T_{1}=T_{2},$ and vulnerability follows the general relationship described by (7). a) The *R*_1_ vs. *R*_2_ plane for the vulnerability saturation parameter, $\psi_{V}=0.5$ and equal surveillance costs for secondary and imported cases, γ=β. The yellow shading indicates the parameter space for which strategy A is cheaper. b) The location of critical lines for different values of *ψ_V_* with γ=β (the greater the value of *ψ_V_*, the faster vulnerability saturates with transmission potential). In each case, strategy A is cost saving for values of (*R*_1_, *R*_2_) below the line and strategy B is cost saving for those above the line. c) The location of critical lines for γ=2β (costs for secondary cases are twice those of imported cases). d) The location of critical lines for γ=5β. (For interpretation of the references to color in this figure legend, the reader is referred to the web version of this article.)

Fig. 4 indicates for which values of *R*_1_ and *R*_2_, the different strategies are to be preferred in terms of costs on the assumption that $T_{1}=T_{2}=T$ and based on the general relationship of *V* to *R* given in Eq. (7). Fig. 4(a) gives the decision rule for one specific set of parameter values. Fig. 4(b)–(d) illustrate the effects of changing the saturation parameter, *ψ_V_*, and the ratio of the surveillance costs of secondary cases to imported cases, *γ:β*.

In general, at low values of *R*, strategy A is favoured, and at high values of *R*, strategy B is favoured. As *ψ_V_* increases, corresponding to a faster saturation of vulnerability with transmission potential, there is an increase in the proportion of settings in which strategy B is cost saving. Increasing the ratio *γ:β* makes it more likely that strategy B will be cheaper while decreasing *γ:β* makes strategy A more attractive — because the number of secondary cases depends strongly on the receptivity of the sink zone. However these effects are relatively small and the indifference curves are rather insensitive to the ratio of *γ* to *β*.

#### 3.2.4 Varying duration of intensified measures

**Fig. 5:**
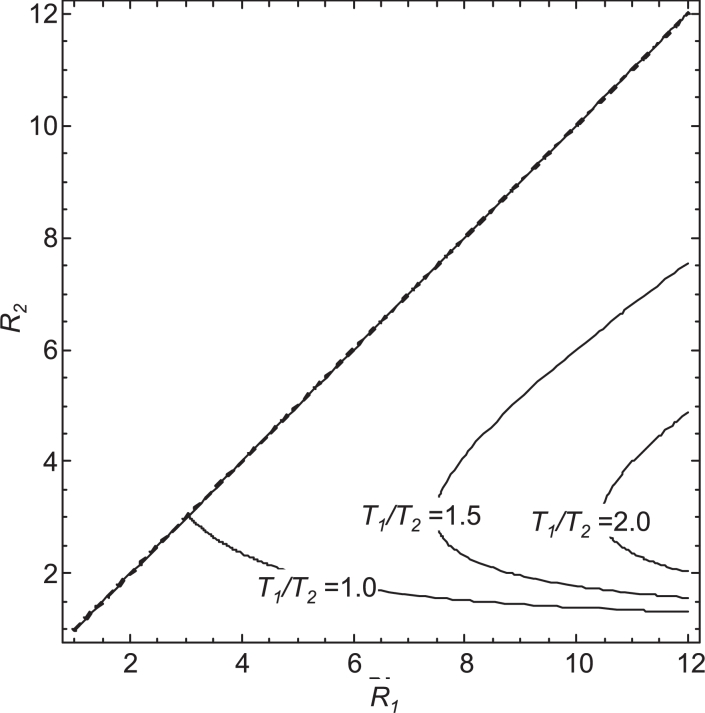
Indifference curves indicating transmission potentials at which the costs are equivalent for the two strategies assuming the required duration of intensified measures increases with transmission, *T*_1_ ≥ *T*_2_, and vulnerability follows the general relationship described by (7). The location of critical lines is shown for different values of the ratio of the required durations of intensified measures, *T*_1_/*T*_2_, with the vulnerability saturation parameter, $\psi_{V}=0.5$ and equal costs for secondary and imported cases, γ=β. In each case, strategy A is cost saving for values of (*R*_1_, *R*_2_) to the left of the line and strategy B is cost saving for values to the right of the line.

Fig. 5 extends the analysis of costs to the case where the required duration of intensified measures varies with transmission potential with an unspecified function, while vulnerability relates to the transmission potential with the general exponential form in (7). The critical lines in the plot of *R*_2_ against *R*_1_ delimiting parameter sets where strategy A is cost saving (to the left of the critical line) move to the right of the plot as *T*_1_/*T*_2_ increases, indicating that if the required duration of the elimination program is greater in the high transmission area, strategy A is cost-saving in more combinations of transmission settings.

**Fig. 6:**
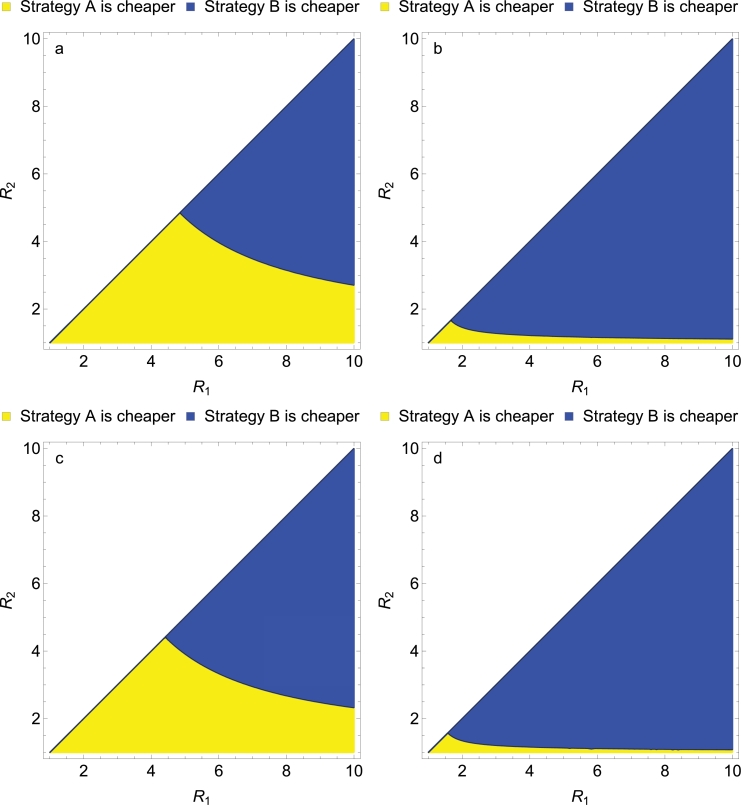
Indifference curves indicating transmission potentials at which the costs are equivalent for the two strategies assuming the required duration of intensified measures, *T*, follows the general relationship given in (8) and vulnerability, *V*, follows the general relationship described by (7). The yellow shading indicates the parameter space for which strategy A is cheaper. a) The costs of secondary cases is equal to that of imported cases, γ=β. The vulnerability saturation parameter $\psi_{V}=0.5$. The saturation parameter for the required duration of intensified measures $\psi_{T}=0.5$. b) γ=β. $\psi_{V}=5$. $\psi_{T}=5$. c) γ=10β (secondary cases cost ten times as much as imported cases). $\psi_{V}=0.5$. $\psi_{T}=0.5$. d) γ=10β. $\psi_{V}=5$. $\psi_{T}=5$. Higher values for *ψ_V_* (respectively *ψ_T_*) correspond to a faster saturation of vulnerability (respectively duration of intensified measures) with transmission. (For interpretation of the references to color in this figure legend, the reader is referred to the web version of this article.)

If the relationship between the duration of intensified measures and the transmission potential follows the general relationship given in (8), then the strategy that minimises costs can be established by substituting the formulae for *f_V_*(*R_i_*) and *f_T_*(*R_i_*) from Eqs. (7) and (8) respectively, into Eq. (17) and simplifying. This gives the general condition for strategy A to be cost saving if

(23) $$\begin{array}{ccc} & & \frac{\left(1-\exp\left(-\psi_{V}\left(R_{1}-1\right)\right)\right)\left(1-\exp\left(-\psi_{T}\left(R_{1}-1\right)\right)\right)\left(\beta +\gamma R_{2}\right)}{\left(1-\exp\left(-\psi_{V}\left(R_{2}-1\right)\right)\right)\left(1-\exp\left(-\psi_{T}\left(R_{2}-1\right)\right)\right)\left(\beta +\gamma R_{1}\right)}\\ & & >1, \end{array}$$

as shown in more detail in Appendix I. Unlike the corresponding analysis of burden, this does not simplify to any simple practical rule, but general trends can be extracted from numerical examples (Fig. 6). If the required duration to interrupt transmission saturates quickly with transmission potential, targeting the low transmission site first (strategy B) is favoured (Fig. 6(b) and (d) compared to Fig. 6(a) and (c)). Similarly, if vulnerability saturates quickly with transmission potential, then targeting the low transmission site is favoured (Fig. 6(b) and (d) compared to Fig. 6(a) and (c)). This is because as vulnerability and duration of intensified measures saturate with transmission potential, the two settings approach the situation with equal durations of intensified measures and equal vulnerability considered above (Section 3.2.2) where strategy B is favoured. Also, if the relative health systems costs of secondary cases (to imported cases) is high, targeting the low transmission site first (strategy B) is favoured (Fig. 6(c) and (d) compared to Fig. 6(a) and (b)).

### 3.3 Net monetary benefit

Where the objectives of minimising disease burden and minimising costs agree on which is the best strategy, this strategy clearly dominates. In the general case, it is possible to combine information on costs and health benefits by assigning a threshold value (*λ*) corresponding to the amount of money that the program is willing to pay for averting one unit of burden of disease. The net monetary benefit of strategy A, in the absence of discounting is then

(24) M=λΔE−ΔC.

This provides an unambiguous decision rule, where the strategy A is favoured if *M* > 0. Substituting Eqs. (11) and (12) in (24) provides

(25) $$M=\lambda \left(B_{1}T_{2}-B_{2}T_{1}\right)-V_{1}T_{2}\left(\beta +\gamma R_{1}\right)+V_{2}T_{1}\left(\beta +\gamma R_{2}\right).$$

The condition for this to be positive (i.e. for strategy A to be preferred within the net benefit framework, with initial intensification in the higher burden area) is then

(26) $$\lambda \left(B_{1}T_{2}-B_{2}T_{1}\right)>\left(V_{1}T_{2}\left(\beta +\gamma R_{1}\right)-V_{2}T_{1}\left(\beta +\gamma R_{2}\right)\right).$$

In low transmission settings with equal durations of elimination programs, there is no trade-off between reducing burden and costs, since both are minimised by targeting the high transmission site first (strategy A), so this strategy dominates. For all other situations the specific values or ranking of the model parameters matter.

The simplest such situation arises with the high transmission case with $T_{1}=T_{2}=T,$ where the health benefits are greater with strategy A, but the costs are also higher, so the preferred strategy depends on trading off health benefits and costs. From Eqs. (20) and (25),

(27) $$M=\left(\lambda \left(B_{1}-B_{2}\right)-\gamma V\left(R_{1}-R_{2}\right)\right)T,$$

leading to the conclusion that strategy B should be favoured if

(28) $$\frac{\left(B_{1}-B_{2}\right)}{\left(R_{1}-R_{2}\right)}<\frac{\gamma V}{\lambda }.$$

If both populations have high transmission initially then, in the limit, burden is independent of transmission intensity, *B*_1_ ≈ *B*_2_ (Fig. 2), and the left hand side of the equation is close to zero. Since the right hand side is always positive, this implies that strategy B is likely to be the most attractive, i.e., the relatively low transmission population should be addressed first. This is because strategy B reduces the number of secondary cases during the second phase of the program (because the imported cases are in the low transmission area).

### 3.4 Summary of the results

When all areas involved have low transmission initially, so that burden and vulnerability are proportional to *R*, and the duration of the intensified measures is independent of initial transmission intensity, the general rule of targeting the higher transmission areas first applies to elimination programs aiming to minimise either total disease burden or costs.

If transmission is initially high in some or all of the zones, or the duration of the intensified measures depends on *R*, then the analysis becomes more complex, and neither strategy dominates. Many specific parameter sets lead to trade-offs between minimising burden and costs, but overall the results can be summarised by the general statements in Table 4.

**Table 4:** Factors favouring Strategy A, i.e., elimination first in the area with higher transmission instead of the targeting the lower transmission area first.

	Priority is to minimise disease burden over time		Priority is to minimise costs over time
1.	Required duration of intensified control measures until transmission is interrupted is independent of transmission potential, *R*.	1.	Required duration of intensified control measures until transmission is interrupted is strongly correlated with *R*.
2.	Annual burden is strongly correlated with *R*.	2.	Vulnerability is strongly correlated with *R*.
		3.	Low transmission levels in both sites.
		4.	Low cost of secondary cases relative to primary cases.

## 4 Discussion and conclusions

Currently there are large investments and efforts to further control malaria burden and move towards elimination in many geographic areas. The strategies to achieve elimination will depend on intense control, focussed efforts to halt and prevent transmission, and improved surveillance and response in existing health systems. Economic analysis can be useful to guide strategic thinking concerning ordering of zones targeted for elimination when the zones have different levels of disease and transmission potential.

In this paper we developed and analysed a mathematical model to understand the implications of prioritisation of different strategies of elimination of neighbouring zones with malaria. The results, summarized in Table 4, indicate general principles that may be applicable at different spatial scales across the whole range of malaria transmission intensities. If all areas involved have low transmission initially, and the duration of intensified control needed to interrupt transmission is the same in all areas, the area with the highest initial level of transmission should be prioritised to minimise total disease burden and/or costs. The mathematics is scale-free and applies whether the areas are small hamlets or islands, districts or countries. The key results extend to any number of different areas, but the models would need significant extension to allow for arbitrary patterns of case exportation and importation.

When some of the areas have high transmission, or the equal duration assumption does not hold, the decision is more complicated. In such cases the strength of the relationship between *T* (time to reduce incidence to negligible levels) and *R* (transmission potential) affects the ordering preference based on burden or on costs in converse ways (Table 4), potentially leading to the need to trade-off these considerations against each other.

As a general rule, we allowed the prevalence (and consequently vulnerability, *V*), the burden, *B*, and the time to elimination, *T* to saturate exponentially with the transmission potential, *R*. This is a reasonable approximation for prevalence and burden, as seen for example in bifurcation diagrams for standard susceptible-infectious-susceptible (SIS) ordinary differential equation (ODE) models. We additionally assumed that the saturation parameters for vulnerability and burden are the same for both areas. This is also reasonable but there may be small differences in the saturation parameters if the degree of seasonality is substantially different between the two areas (for example if one area is highly seasonal while the other is relatively flat) (Stuckey et al., 2014).

However, assuming an exponentially saturating relationship between *T* and *R* remains a simplifying assumption, considering the heterogeneity of contexts and strategies in elimination programs. We use an exponential function because the relationship should be monotonic and if elimination is feasible, it is likely that the time to elimination will saturate as a function of transmission potential. We expect that *T* is considerably longer than the time required for residual infections to clear once transmission has been interrupted (Smith and Hay, 2009). In a stable environment, the maximal impact on transmission of a malaria intervention program occurs shortly after scale-up (Smith and Schapira, 2012) but the trajectories of most recent elimination programs do not correspond to the GMEP model with fixed *T* (Macdonald, Göckel, 1964, Pampana, 1963). Many factors work to make *T* longer and more irregular in contemporary programs: tropical areas in most cases have entomological characteristics slowing or blunting the effectiveness of available vector control measures; the populations needing coverage may be large; human ecology, for example population movement, may constrain intervention effectiveness; and long lasting insecticidal nets, if used as the main transmission-reducing intervention, may achieve more gradual scale-up of impact than indoor residual spraying. Furthermore, health system heterogeneities may greatly increase the time needed to interrupt transmission, which depends not only on vector control, but also on surveillance as part of the intensified measures package. Accounting for the uncertainty in the *T* may be in incorporated into program planning but would require substantially more complicated stochastic models. Nonetheless, given the profound influence of variations in *T*, demonstrated here, it would be rational for elimination programs to assess *T* as carefully as possible in different zones, based on experience and knowledge about determinants of intervention effectiveness, to better clarify the relationship between *T* and *R*.

Other factors, especially costs, also clearly depend on local information. When initial transmission and prevalence are high, surveillance costs may be minimised if the lower reproduction number zone is targeted first, since vulnerability saturates with level of transmission so that introduced cases are equally frequent whichever ordering is chosen, while secondary infection rates depend on the local reproduction number. The ratio between surveillance costs for primary and secondary cases is then relevant to the choice of strategy, and a field-determined value of this would be needed for a full quantitative analysis of any specific situation. Population sizes and per capita costs may also vary between locations. However, to identify important principles, we also made the simplifying assumption that the two zones have similar population sizes and the same base costs of setting up the surveillance system.

In reality, there is also likely to be temporal overlap between intensification of control in different zones. Temporal overlap reduces the marginal cost of surveillance proportionately to the reduction in overall duration, but this should not change the preferred ordering, because the mathematics does not change if the import/export of cases during the overlap period is small. In the limiting case, when transmission is interrupted everywhere in the world (and complete eradication is achieved) simultaneously, the surveillance period post-elimination and its costs are zero.

The most critical assumption here is that elimination is feasible. There is always more uncertainty about a strategy with a specific endpoint (elimination) than to a less specific one (control), and there is a need for decision rules allowing for this uncertainty (which go beyond the scope of this paper). Spatially progressive elimination can be an appropriate eradication strategy for infections with patchy distributions and long generation times (like onchocerciasis) or where intervention strategies lead to long-term protection of humans from infection (as with smallpox vaccination); but it is unclear under which circumstances this will work for malaria, where neither of these conditions hold. In most endemic settings, the spatial distribution of malaria is widespread, malaria parasites have a short generation time (about two months in the case of *Plasmodium falciparum* (Huber et al., 2016) and the effects of most interventions are of a relatively short duration, so transmission will recover rapidly if intervention deployment ceases.

In many settings, the rationale for spatially progressive elimination therefore depends critically on the assumption that if transmission is interrupted, it is unlikely to re-establish itself (i.e., the malaria free state is asymptotically stable — or ‘sticky’ (Smith et al., 2013). This can be the case if an effective surveillance system is in place and infections become more detectable as transmission is brought down because of loss of clinical immunity, and/or the vectorial capacity is low from the beginning. It may also be unlikely for transmission to resume if malaria elimination is accompanied by changes in housing or lifestyles that permanently reduce vectorial capacity. If such changes do not occur, then the risk of reintroduction can be contained by indefinite maintenance of pro-active vector control. In most situations, the costs of this would be unacceptably high, so ‘stickiness’ depends on changes in the human and vector ecology that are contingent on vector biology, economic development and equity, and hence outside the control of the health system. However, analysis of countries that are either on the path to elimination or have already achieved elimination has shown that while resurgence is likely to occur in countries that have not achieved elimination, it is unlikely to occur after elimination has been reached (Cohen, Smith, Cotter, Ward, Yamey, Sabot, Moonen, 2012, Feachem, Phillips, Targett, 2009, Smith, Cohen, Chiyaka, Johnston, Gething, Gosling, Buckee, Laxminarayan, Hay, Tatem, 2013). Smith et al. (2013) state:

What is surprising, however, is that of the 50 elimination programmes identified as successful, only four (8%) were found to have experienced resurgence, despite continued importation over many years. Moreover of these four, two eliminated malaria and are malaria-free once again. These 50 countries still have competent vectors and few have ongoing transmission-lowering activities.

Therefore, it is likely that surveillance programs will be able to maintain elimination after it has been achieved.

Rational decision-making about prioritising low or high transmission areas in an elimination programme may be straightforward under certain conditions: if transmission is initially relatively low in all areas, if the disease burden is strongly correlated with level of transmission, while expected time to elimination is not, then the high transmission area should be prioritised. Otherwise, strategic planning would benefit from a detailed assessment of patterns of vulnerability and surveillance costs that relaxes the assumptions made in this analysis.

## Appendix A Preliminary analysis

Lemma 1 *Let*

(A.1) $$f\left(x\right)=\left(e^{ax}-1\right)\left(e^{a/x}-1\right)$$

*with a* > 0 *and domain* D={x∈R|x>0}*. Then f*(*x*) *has a global minimum at 1, that is,*

(A.2) f(1)≤f(x)∀x∈D.

Proof $f\left(x\right)=\left(e^{ax}-1\right)\left(e^{a/x}-1\right)$ has a global minimum at x=1 if and only if

(A.3a) $$\begin{array}{cc} f(x)-1 & =\left(e^{ax}-1\right)\left(e^{a/x}-1\right)-1 \end{array}$$

(A.3b) $$\begin{array}{cc} & =e^{ax+a/x}-e^{ax}-e^{a/x}, \end{array}$$

has a global minimum at x=1. For notational convenience we redefine

(A.4) $$f\left(x\right)=e^{ax+a/x}-e^{ax}-e^{a/x}.$$

To prove that *f*(*x*) has a global minimum at x=1, we will show that:

(i) $f^{\prime }\left(1\right)=0$
(ii) *f*′(*x*) > 0 for *x* > 1
(iii) *f*′(*x*) < 0 for 0 < *x* < 1,

where *f*′(*x*) denotes the derivative of *f*(*x*) with respect to *x*,

(A.5) $$f^{\prime }\left(x\right)=\left(a-\frac{a}{x^{2}}\right)e^{ax+a/x}-ae^{ax}+\frac{a}{x^{2}}e^{a/x}.$$

Substituting x=1 shows that $f^{\prime }\left(1\right)=0$ so condition (i) is satisfied. To prove conditions (ii) and (iii), we use the change of variables,

(A.6) u=ln(x),

with u∈R and $x=e^{u},$ to define

(A.7) $$g\left(u\right)=f\left(e^{u}\right)=e^{a(e^{u}+e^{-u})}-e^{ae^{u}}-e^{ae^{-u}}.$$

Since *u* is a monotonically increasing function of *x*, with x=1 corresponding to u=0, statement (ii) is equivalent to showing that *g*′(*u*) > 0 for *u* > 0 and statement (iii) is equivalent to showing that *g*′(*u*) < 0 for *u* < 0, where *g*′(*u*) denotes the derivative of *g*(*u*) with respect to *u*,

(A.8) $$g^{\prime }\left(u\right)=ae^{u}\left(e^{a(e^{u}+e^{-u})}-e^{ae^{u}}\right)-ae^{-u}\left(e^{a(e^{u}+e^{-u})}-e^{ae^{-u}}\right).$$

We introduce the function

(A.9) $$\begin{array}{cc} h(u) & =\frac{ae^{u}\left(e^{a(e^{u}+e^{-u})}-e^{ae^{u}}\right)}{ae^{-u}\left(e^{a(e^{u}+e^{-u})}-e^{ae^{-u}}\right)}\\ & =e^{2u}\frac{e^{ae^{u}}\left(e^{ae^{-u}}-1\right)}{e^{ae^{-u}}\left(e^{ae^{u}}-1\right)}, \end{array}$$

so that *h*(*u*) > 1 is equivalent to *g*′(*u*) > 0 and *h*(*u*) < 1 is equivalent to *g*′(*u*) < 0. We note here that

(A.10) $$e^{\xi }-1=\xi e^{\xi -c(\xi )},$$

for

(A.11) $$c\left(\xi \right)=\ln\left(\xi \right)-\ln\left(1-e^{-\xi }\right).$$

Then using the above substitution (A.11), with $\xi =ae^{-u}$ in the numerator of *h*(*u*) and $\xi =ae^{u}$ in the denominator of *h*(*u*), and simplifying,

(A.12) $$\begin{array}{cc} h(u) & =\frac{e^{-c(ae^{-u})}}{e^{-c(ae^{u})}}\\ & =e^{c\left(ae^{u}\right)-c\left(ae^{-u}\right)}. \end{array}$$

Introducing the function

(A.13) $$j\left(u\right)=c\left(ae^{u}\right)-c\left(ae^{-u}\right),$$

we see that if j(u)=0, then h(u)=1; if *j*(*u*) > 0, then *h*(*u*) > 1; and if *j*(*u*) < 0, then *h*(*u*) < 1. We expand

(A.14) $$j\left(u\right)=2u+\ln\left(1-e^{-ae^{-u}}\right)-\ln\left(1-e^{-ae^{u}}\right),$$

see that j(0)=0, and differentiate

(A.15) $$j^{\prime }\left(u\right)=2-\frac{v_{-}e^{-v_{-}}}{1-e^{-v_{-}}}-\frac{v_{+}e^{-v_{+}}}{1-e^{-v_{+}}},$$

where

(A.16a) $$\begin{array}{cc} v_{-} & =ae^{-u}, \end{array}$$

(A.16b) $$\begin{array}{cc} v_{+} & =ae^{u}. \end{array}$$

Noting that, $\xi <e^{\xi }-1,$ for *ξ* ≠ 0,

(A.17) $$\begin{array}{cc} j^{\prime }\left(u\right) & >2-\frac{\left(e^{v_{-}}-1\right)e^{-v_{-}}}{1-e^{-v_{-}}}-\frac{\left(e^{v_{+}}-1\right)e^{-v_{+}}}{1-e^{-v_{+}}} \end{array}$$

(A.18) $$\begin{array}{cc} & =0. \end{array}$$

Since j(0)=0 and *j*(*u*) is monotonically increasing for all u∈R, for *u* < 0, *j*(*u*) < 0, and for *u* > 0, *j*(*u*) > 0. Therefore, for *u* > 0, *h*(*u*) > 1, *g*′(*u*) > 0, and correspondingly, *f*′(*x*) > 0 for *x* > 1 so condition (ii) is satisfied. Similarly, for *u* < 0, *h*(*u*) < 1, *g*′(*u*) < 0, and correspondingly, *f*′(*x*) < 0 for 0 < *x* < 1 so condition (iii) is satisfied. □
Lemma 2 *For all* φ > 0, *θ* > 0, *x* > 0 *and y* > 0,

(A.19) $$e^{\varphi x+\theta y}+e^{-\varphi y}+e^{-\theta x}>e^{-(\varphi y+\theta x)}+e^{\varphi x}+e^{\theta y}.$$

Proof We rearrange the inequality to be proved (A.19) as

(A.20) $$e^{\varphi x+\theta y}-e^{\varphi x}-e^{\theta y}>e^{-\varphi y-\theta x}-e^{-\varphi y}-e^{-\theta x}.$$

We introduce the change of variables,

(A.21a) $$\begin{array}{cc} s & =\varphi y+\theta x, \end{array}$$

(A.21b) $$\begin{array}{cc} ɛ & =\frac{\varphi y}{\varphi y+\theta x}, \end{array}$$

(A.21c) $$\begin{array}{cc} t & =\frac{\varphi x}{\theta y}, \end{array}$$

with *s* > 0, *t* > 0, and 0 < ε < 1. Then

(A.22a) $$\begin{array}{cc} \varphi x & =\sqrt{ɛ(1-ɛ)}s\sqrt{t}, \end{array}$$

(A.22b) $$\begin{array}{cc} \varphi y & =ɛs, \end{array}$$

(A.22c) $$\begin{array}{cc} \theta x & =(1-ɛ)s, \end{array}$$

(A.22d) $$\begin{array}{cc} \theta y & =\sqrt{ɛ(1-ɛ)}s\frac{1}{\sqrt{t}}. \end{array}$$

Using the change of variables, (A.21), the inequality to be proved (A.20) becomes

(A.23) $$\begin{array}{ccc} & & e^{\sqrt{ɛ(1-ɛ)}s\left(\sqrt{t}+1/\sqrt{t}\right)}-e^{\sqrt{ɛ(1-ɛ)}s\sqrt{t}}-e^{\sqrt{ɛ(1-ɛ)}s\left(1/\sqrt{t}\right)}\\ & & >e^{-s}-e^{-ɛs}-e^{-(1-ɛ)s}, \end{array}$$

which is equivalent to

(A.24) $$\begin{array}{ccc} & & \left(e^{\sqrt{ɛ(1-ɛ)}s\sqrt{t}}-1\right)\left(e^{\sqrt{ɛ(1-ɛ)}s\left(1/\sqrt{t}\right)}-1\right)\\ & & >\left(e^{-ɛs}-1\right)\left(e^{-(1-ɛ)s}-1\right). \end{array}$$

Applying Lemma 1 to the left hand side of (A.24) with $a=\sqrt{ɛ(1-ɛ)}s$ and $x=\sqrt{t},$ implies

(A.25) $${\left(e^{\sqrt{ɛ(1-ɛ)}s}-1\right)}^{2}\leq \left(e^{\sqrt{ɛ(1-ɛ)}s\sqrt{t}}-1\right)\left(e^{\sqrt{ɛ(1-ɛ)}s\left(1/\sqrt{t}\right)}-1\right).$$

Therefore to prove inequality (A.24), it is sufficient to show that

(A.26) $${\left(e^{\sqrt{ɛ(1-ɛ)}s}-1\right)}^{2}>\left(e^{-ɛs}-1\right)\left(e^{-(1-ɛ)s}-1\right),$$

which is equivalent to proving

(A.27) $$\frac{{\left(e^{\sqrt{ɛ(1-ɛ)}s}-1\right)}^{2}}{\left(e^{-ɛs}-1\right)\left(e^{-(1-ɛ)s}-1\right)}>1.$$

Using the inequalities

(A.28a) $$\begin{array}{cc} e^{x}-1 & >x+\left(1/2\right)x^{2}\;\;\text{for}\;x>0, \end{array}$$

(A.28b) $$\begin{array}{cc} e^{x}-1 & <x+\left(1/2\right)x^{2}\;\;\text{for}\;x<0, \end{array}$$

we see that

(A.29) $$\begin{array}{cc} & \frac{{\left(e^{\sqrt{ɛ(1-ɛ)}s}-1\right)}^{2}}{\left(e^{-ɛs}-1\right)\left(e^{-(1-ɛ)s}-1\right)}\\ & \;>\frac{{\left(\sqrt{ɛ(1-ɛ)}s+\left(1/2\right)ɛ\left(1-ɛ\right)s^{2}\right)}^{2}}{\left(-ɛs+\left(1/2\right){ɛ}^{2}s^{2}\right)\left(-\left(1-ɛ\right)s+\left(1/2\right){\left(1-ɛ\right)}^{2}s^{2}\right)}\\ & \;=\frac{ɛ\left(1-ɛ\right)s^{2}+\left(1/4\right){ɛ}^{2}{\left(1-ɛ\right)}^{2}s^{4}+{\left(ɛ\left(1-ɛ\right)\right)}^{3/2}s^{3}}{ɛ\left(1-ɛ\right)s^{2}+\left(1/4\right){ɛ}^{2}{\left(1-ɛ\right)}^{2}s^{4}-\left(1/2\right)ɛ\left(1-ɛ\right)s^{3}}\\ & \;>1. \end{array}$$

 □

## Appendix B Analysis 1: Equal time and higher burden

The main assumptions are:

(B.1a) $$\begin{array}{cc} T_{1} & =T_{2}, \end{array}$$

(B.1b) $$\begin{array}{cc} B_{1} & >B_{2}. \end{array}$$

The reduction in burden due to targeting site 1 first is

(B.2) $$\begin{array}{ccc} \Delta E & = & B_{1}T_{2}-B_{2}T_{1}\\ & = & \left(B_{1}-B_{2}\right)T\\ & > & 0. \end{array}$$

Therefore, Strategy A always reduces burden.

## Appendix C Analysis 2: Higher time and higher burden

The main assumptions are:

(C.1a) $$\begin{array}{cc} T_{1} & >T_{2}, \end{array}$$

(C.1b) $$\begin{array}{cc} B_{1} & >B_{2}. \end{array}$$

The reduction in burden due to targeting site 1 first is

(C.2) $$\Delta E=B_{1}T_{2}-B_{2}T_{1}.$$

Therefore, Strategy A reduces burden if and only if

(C.3) $$B_{1}T_{2}>B_{2}T_{1},$$

or equivalently,

(C.4) $$\frac{B_{1}}{B_{2}}>\frac{T_{1}}{T_{2}}.$$

## Appendix D Analysis 3: Saturating time and saturating burden

The main assumptions are:

(D.1a) $$\begin{array}{cc} T_{i} & =T_{\max}\left(1-e^{-\psi_{T}\left(R_{i}-1\right)}\right)\;\;\text{for}\;\;i\in \left\{1,2\right\}, \end{array}$$

(D.1b) $$\begin{array}{cc} B_{i} & =B_{\max}\left(1-e^{-\psi_{B}\left(R_{i}-1\right)}\right)\;\;\text{for}\;\;i\in \left\{1,2\right\}. \end{array}$$

The reduction in burden due to targeting site 1 first is

(D.2) $$\begin{array}{ccc} \Delta E & = & B_{1}T_{2}-B_{2}T_{1}\\ & = & B_{\max}T_{\max}[\left(1-e^{-\psi_{B}\left(R_{1}-1\right)}\right)\left(1-e^{-\psi_{T}\left(R_{2}-1\right)}\right)\\ & & -\left(1-e^{-\psi_{B}\left(R_{2}-1\right)}\right)\left(1-e^{-\psi_{T}\left(R_{1}-1\right)}\right)]. \end{array}$$

We first assume *ψ_B_* > *ψ_T_*, and use the change of variables,

(D.3a) $$\begin{array}{cc} y & =R_{2}-1, \end{array}$$

(D.3b) $$\begin{array}{cc} x & =R_{1}-R_{2}, \end{array}$$

(D.3c) $$\begin{array}{cc} \varphi & =\psi_{T}, \end{array}$$

(D.3d) $$\begin{array}{cc} \theta & =\psi_{B}-\psi_{T}, \end{array}$$

with *x* > 0 (since *R*_1_ > *R*_2_), *y* > 0, φ > 0, and *θ* > 0, to rewrite (D.2) as

(D.4) $$\begin{array}{ccc} \Delta E & = & B_{\max}T_{\max}[\left(1-e^{-(\varphi +\theta )(x+y)}\right)\left(1-e^{-\varphi y}\right)\\ & & \;-\left(1-e^{-(\varphi +\theta )y}\right)\left(1-e^{-\varphi (x+y)}\right)]. \end{array}$$

We rewrite (D.4) after some algebraic manipulations as

(D.5) $$\begin{array}{ccc} \Delta E & = & B_{\max}T_{\max}e^{-(\varphi x+\varphi y+\theta y)}[\left(e^{-(\varphi y+\theta x)}+e^{\varphi x}+e^{\theta y}\right)\\ & & \;-\left(e^{\varphi x+\theta y}+e^{-\varphi y}+e^{-\theta x}\right)]. \end{array}$$

Applying Lemma 2 (see Appendix A) to (D.5) we see, that for *ψ_B_* > *ψ_T_*,

(D.6) ΔE<0,

so strategy B reduces burden.

A similar calculation for *ψ_T_* > *ψ_B_*, with the change of variables,

(D.7a) $$\begin{array}{cc} y & =R_{2}-1, \end{array}$$

(D.7b) $$\begin{array}{cc} x & =R_{1}-R_{2}, \end{array}$$

(D.7c) $$\begin{array}{cc} \zeta & =\psi_{B}, \end{array}$$

(D.7d) $$\begin{array}{cc} \nu & =\psi_{T}-\psi_{B}, \end{array}$$

with *x* > 0, *y* > 0, *ζ* > 0, and *ν* > 0, shows that

(D.8) $$\begin{array}{ccc} \Delta E & = & B_{\max}T_{\max}e^{-(\zeta x+\zeta y+\nu y)}[\left(e^{\zeta x+\nu y}+e^{-\zeta y}+e^{-\nu x}\right)\\ & & \;-\left(e^{-(\zeta y+\nu x)}+e^{\zeta x}+e^{\nu y}\right)], \end{array}$$

where by Lemma 2,

(D.9) ΔE>0,

so strategy A reduces burden.

## Appendix E Analysis 4: Equal time and linear vulnerability

The main assumptions are:

(E.1a) $$\begin{array}{cc} T_{1} & =T_{2}, \end{array}$$

(E.1b) $$\begin{array}{cc} V_{i} & =\eta \left(R_{j}-1\right)\;\;\text{for}\;\;i,j\in \left\{1,2\right\}\;\text{with}\;i\neq j. \end{array}$$

The additional costs of strategy A are

(E.2) $$\begin{array}{cc} \Delta C & =V_{1}T_{2}\left(\beta +\gamma R_{1}\right)-V_{2}T_{1}\left(\beta +\gamma R_{2}\right)\\ & =\eta \left(R_{2}-1\right)T\left(\beta +\gamma R_{1}\right)-\eta \left(R_{1}-1\right)T\left(\beta +\gamma R_{2}\right)\\ & =\eta T\left(\beta +\gamma \right)\left(R_{2}-R_{1}\right)\\ & <0. \end{array}$$

Therefore, Strategy A is always cheaper.

## Appendix F Analysis 5: Higher time and linear vulnerability

The main assumptions are:

(F.1a) $$\begin{array}{cc} T_{1} & >T_{2}, \end{array}$$

(F.1b) $$\begin{array}{cc} V_{i} & =\eta \left(R_{j}-1\right)\;\;\text{for}\;\;i,j\in \left\{1,2\right\}\;\text{with}\;i\neq j. \end{array}$$

The additional costs of strategy A are

(F.2) $$\begin{array}{cc} \Delta C & =V_{1}T_{2}\left(\beta +\gamma R_{1}\right)-V_{2}T_{1}\left(\beta +\gamma R_{2}\right)\\ & =\eta \left(R_{2}-1\right)T_{2}\left(\beta +\gamma R_{1}\right)-\eta \left(R_{1}-1\right)T_{1}\left(\beta +\gamma R_{2}\right). \end{array}$$

We use the change of variables,

(F.3) $$T_{d}=T_{1}-T_{2},$$

with *T_d_* > 0, and $T_{1}=T_{d}+T_{2}$ to rewrite (F.2) as

(F.4) $$\begin{array}{cc} \Delta C & =\eta T_{2}\left(\beta +\gamma \right)\left(R_{2}-R_{1}\right)-\eta \left(R_{1}-1\right)T_{d}\left(\beta +\gamma R_{2}\right)\\ & <0. \end{array}$$

Therefore, Strategy A is always cheaper.

## Appendix G Analysis 6: Equal time and equal vulnerability

The main assumptions are:

(G.1a) $$\begin{array}{cc} T_{1} & =T_{2}, \end{array}$$

(G.1b) $$\begin{array}{cc} V_{1} & =V_{2}. \end{array}$$

The additional costs of strategy A are

(G.2) $$\begin{array}{cc} \Delta C & =V_{1}T_{2}\left(\beta +\gamma R_{1}\right)-V_{2}T_{1}\left(\beta +\gamma R_{2}\right)\\ & =VT\gamma (R_{1}-R_{2})\\ & >0. \end{array}$$

Therefore, Strategy B is always cheaper.

## Appendix H Analysis 7: Higher time and equal vulnerability

The main assumptions are:

(H.1a) $$\begin{array}{cc} T_{1} & >T_{2}, \end{array}$$

(H.1b) $$\begin{array}{cc} V_{1} & =V_{2}. \end{array}$$

The additional costs of strategy A are

(H.2) $$\begin{array}{cc} \Delta C & =V_{1}T_{2}\left(\beta +\gamma R_{1}\right)-V_{2}T_{1}\left(\beta +\gamma R_{2}\right)\\ & =VT_{2}\left(\beta +\gamma R_{1}\right)-VT_{1}\left(\beta +\gamma R_{2}\right)\\ & =V\beta \left(T_{2}-T_{1}\right)+V\gamma \left(T_{2}R_{1}-T_{1}R_{2}\right). \end{array}$$

The sign of (H.2) depends on parameter values, and we can show with some algebraic manipulations that strategy A is cheaper (*ΔC* < 0) when

(H.3) $$R_{2}>\frac{T_{2}}{T_{1}}\left(R_{1}-\frac{\beta }{\gamma }\left(\frac{T_{1}-T_{2}}{T_{2}}\right)\right).$$

## Appendix I Analysis 8: Saturating time and saturating vulnerability

The main assumptions are:

(I.1a) $$\begin{array}{cc} T_{i} & =T_{\max}\left(1-e^{-\psi_{T}\left(R_{i}-1\right)}\right)\;\;\text{for}\;\;i\in \left\{1,2\right\}, \end{array}$$

(I.1b) $$\begin{array}{cc} V_{i} & =V_{\max}\left(1-e^{-\psi_{V}\left(R_{j}-1\right)}\right)\;\;\text{for}\;\;i,j\in \left\{1,2\right\}\;\text{with}\;i\neq j. \end{array}$$

The additional costs of strategy A are

(I.2) $$\begin{array}{cc} \Delta C & =V_{\max}\left(1-e^{-\psi_{V}\left(R_{2}-1\right)}\right)T_{\max}\left(1-e^{-\psi_{T}\left(R_{2}-1\right)}\right)\left(\beta +\gamma R_{1}\right)\\ & \;-V_{\max}\left(1-e^{-\psi_{V}\left(R_{1}-1\right)}\right)T_{\max}\left(1-e^{-\psi_{T}\left(R_{1}-1\right)}\right)\left(\beta +\gamma R_{2}\right)\\ & =V_{\max}T_{\max}\beta [\left(1-e^{-\psi_{V}\left(R_{2}-1\right)}\right)\left(1-e^{-\psi_{T}\left(R_{2}-1\right)}\right)\left(1+\frac{\gamma }{\beta }R_{1}\right)\\ & \;-\left(1-e^{-\psi_{V}\left(R_{1}-1\right)}\right)\left(1-e^{-\psi_{T}\left(R_{1}-1\right)}\right)\left(1+\frac{\gamma }{\beta }R_{2}\right)]. \end{array}$$

We do not have an explicit analytical expression that relates the sign of *ΔC* to *R*_1_, *R*_2_ and the parameters so we rely on the numerical simulations as shown in the main text.
